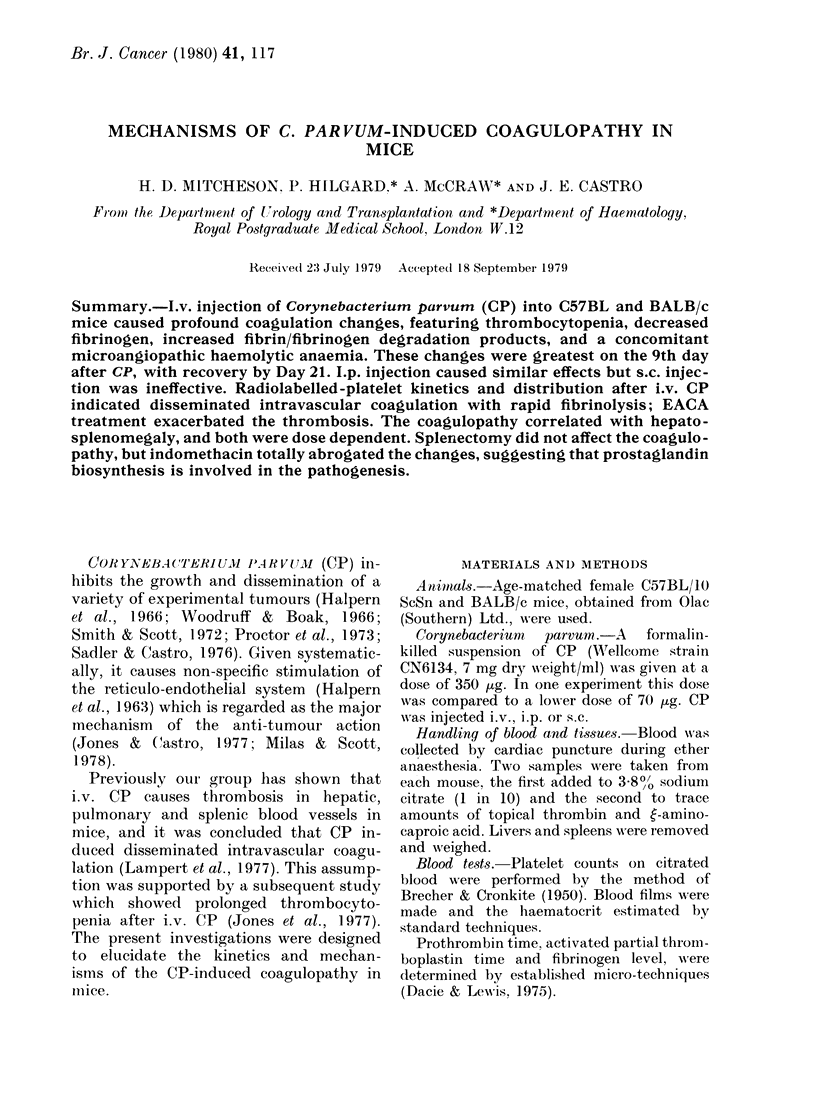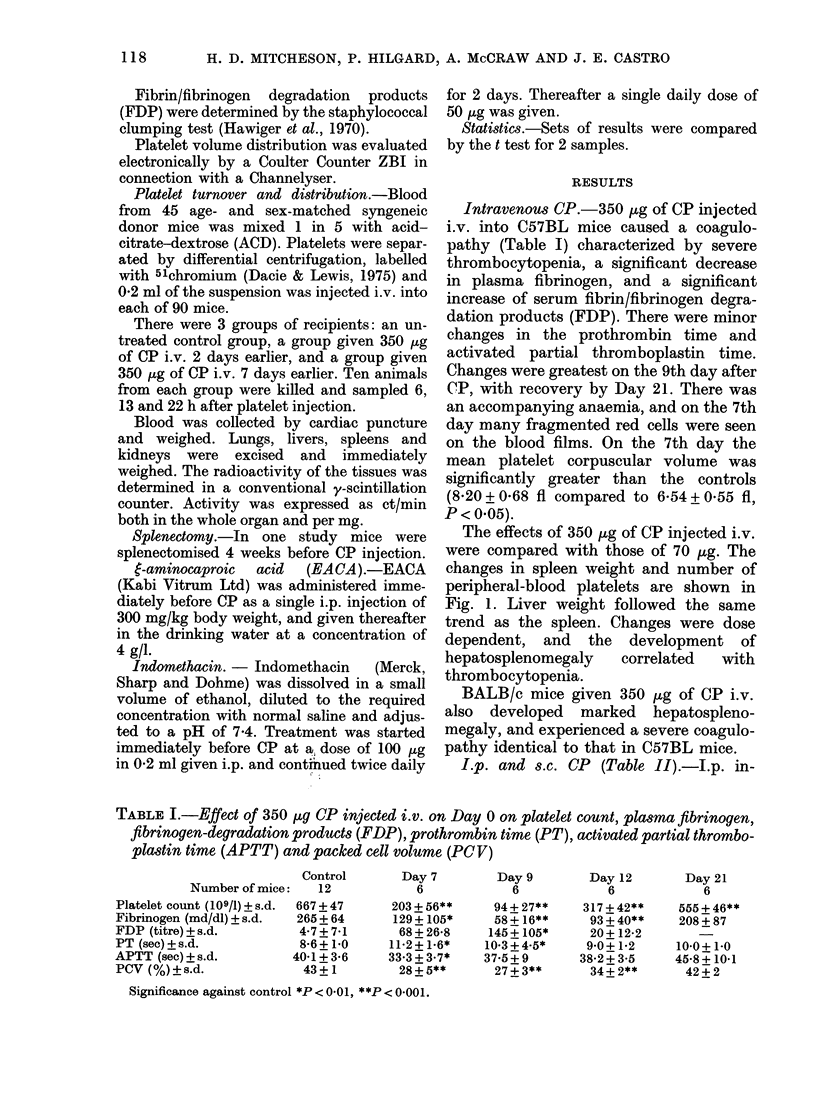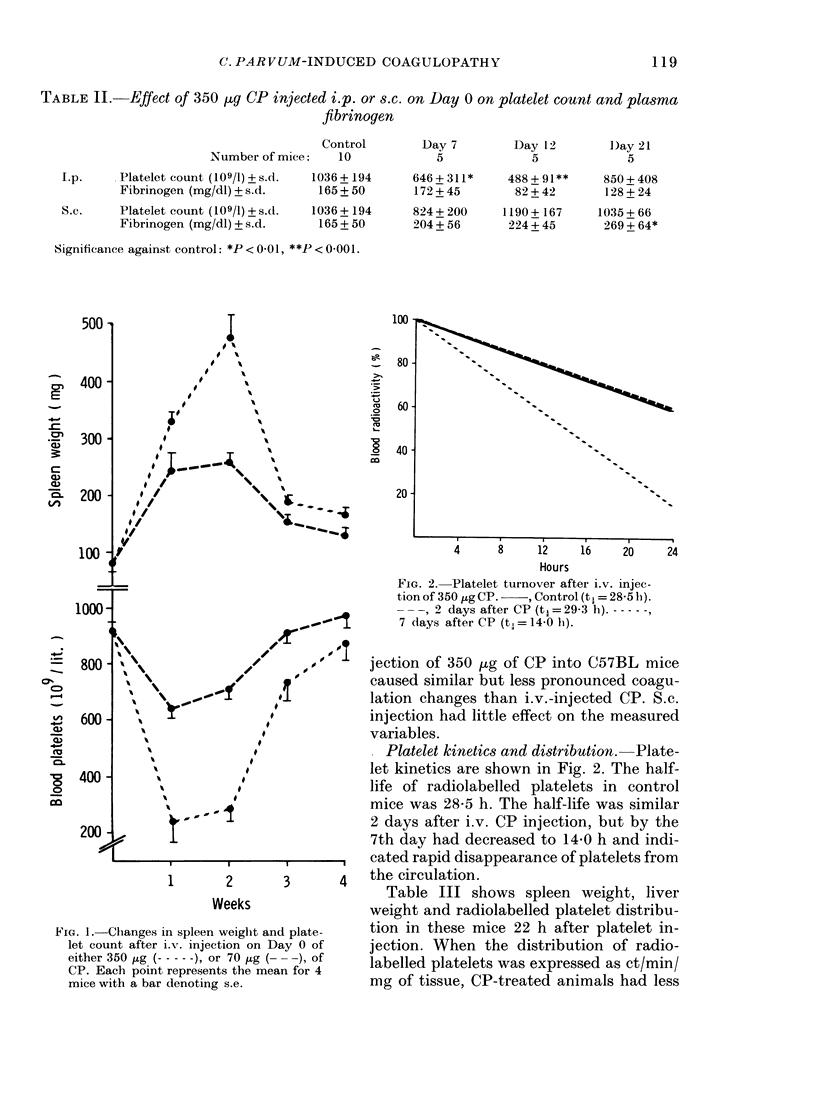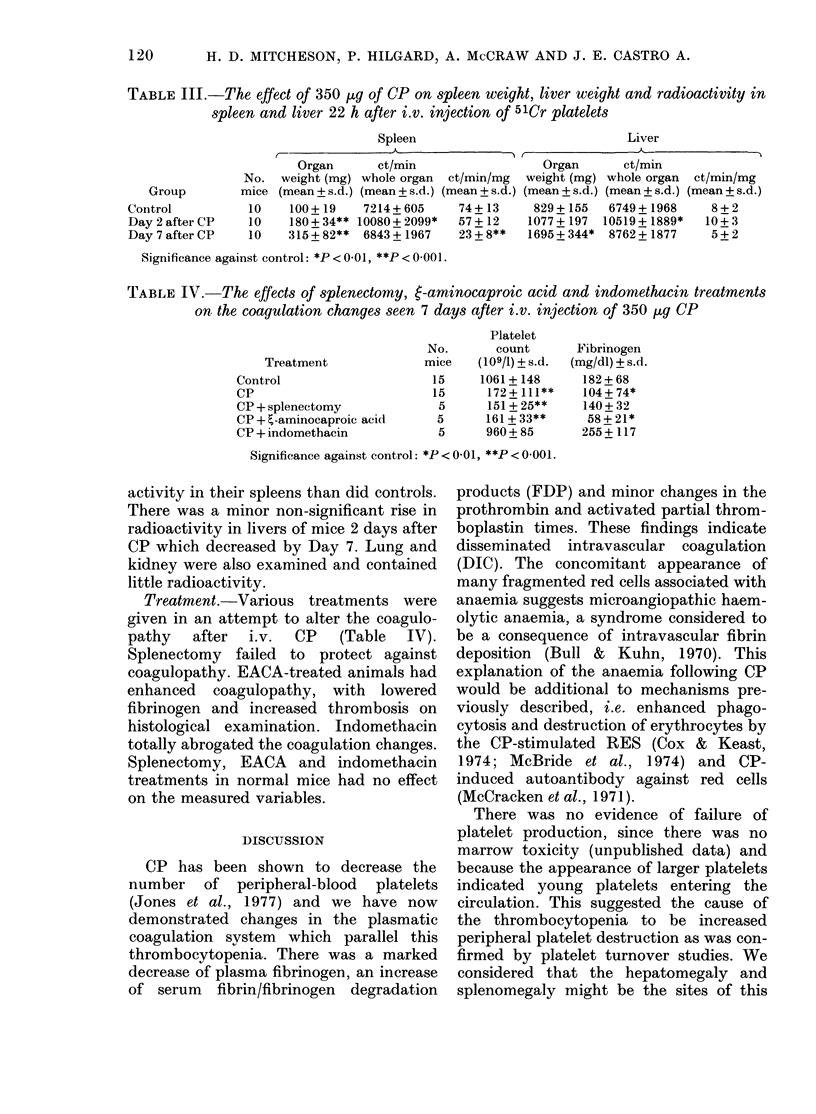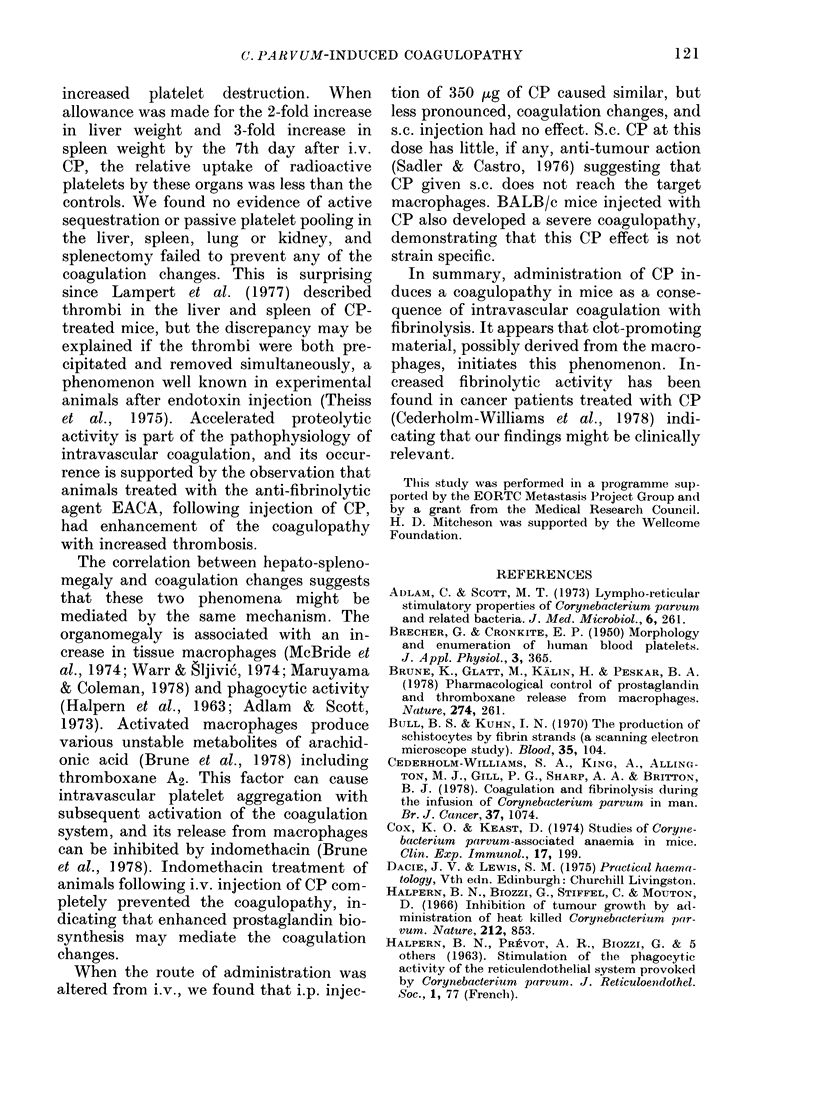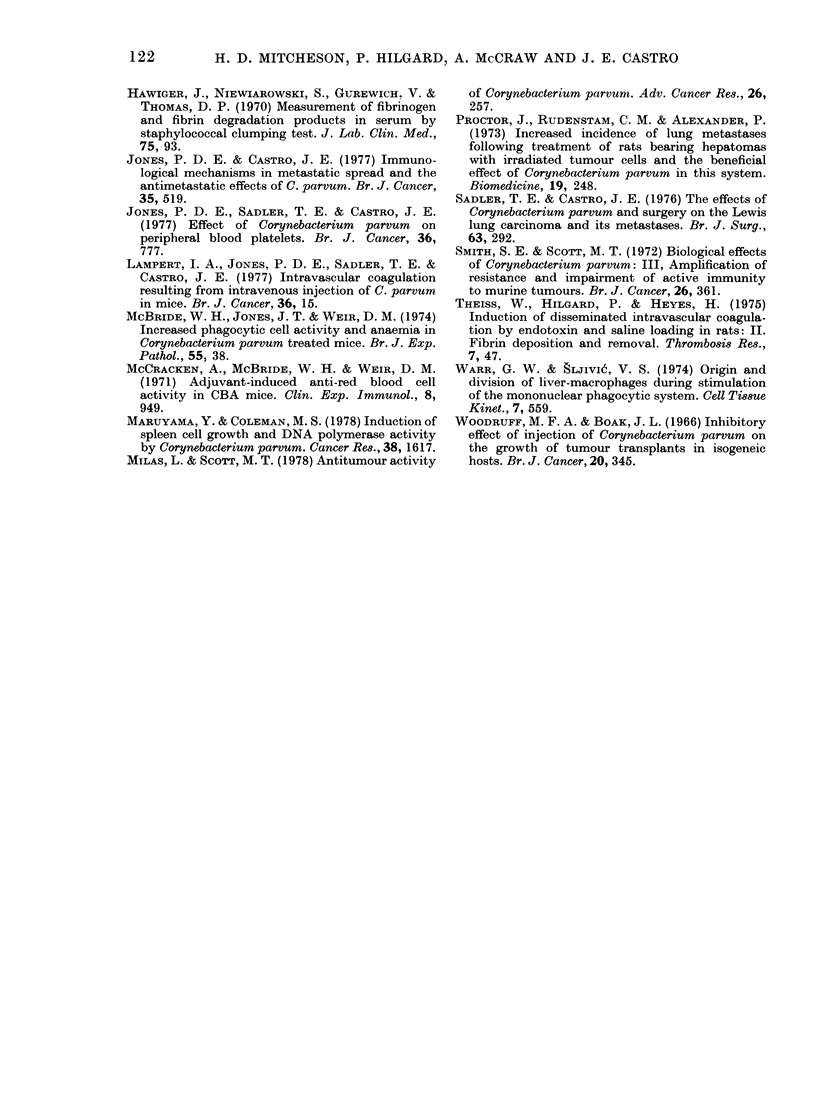# Mechanisms of C. Parvum-induced coagulopathy in mice.

**DOI:** 10.1038/bjc.1980.13

**Published:** 1980-01

**Authors:** H. D. Mitcheson, P. Hilgard, A. McCraw, J. E. Castro

## Abstract

I.v. injection of Corynebacterium parvum (CP) into C57BL and BALB/c mice caused profound coagulation changes, featuring thrombocytopenia, decreased fibrinogen, increased fibrin/fibrinogen degradation products, and a concomitant microangiopathic haemolytic anaemia. These changes were greatest on the 9th day after CP, with recovery by Day 21. I.p. injection caused similar effects but s.c. injection was ineffective. Radiolabelled-platelet kinetics and distribution after i.v. CP indicated disseminated intravascular coagulation with rapid fibrinolysis; EACA treatment exacerbated the thrombosis. The coagulopathy correlated with hepatosplenomegaly, and both were dose dependent. Splenectomy did not effect the coagulopathy, but indomethacin totally abrogated the changes, suggesting that prostaglandin biosynthesis is involved in the pathogenesis.


*Br. J. Cancer* (1980) **41**, 117

### MECHANISMS OF *C. PARVUM*-INDUCED COAGULOPATHY IN MICE

H. D. MITCHESON, P. HILGARD,* A. McCRAW* AND J. E. CASTRO

*From the Department of Urology and Transplantation and *Department of Haematology, Royal Postgraduate Medical School, London W.12*

Received 23 July 1979 Accepted 18 September 1979

**Summary.—I.v. injection of *Corynebacterium parvum* (CP) into C57BL and BALB/c mice caused profound coagulation changes, featuring thrombocytopenia, decreased fibrinogen, increased fibrin/fibrinogen degradation products, and a concomitant microangiopathic haemolytic anaemia. These changes were greatest on the 9th day after *CP*, with recovery by Day 21. I.p. injection caused similar effects but s.c. injection was ineffective. Radiolabelled-platelet kinetics and distribution after i.v. CP indicated disseminated intravascular coagulation with rapid fibrinolysis; EACA treatment exacerbated the thrombosis. The coagulopathy correlated with hepatosplenomegaly, and both were dose dependent. Splenectomy did not affect the coagulopathy, but indomethacin totally abrogated the changes, suggesting that prostaglandin biosynthesis is involved in the pathogenesis.**

*CORYNEBACTERIUM PARVUM* (CP) inhibits the growth and dissemination of a variety of experimental tumours (Halpern *et al.*, 1966; Woodruff & Boak, 1966; Smith & Scott, 1972; Proctor *et al.*, 1973; Sadler & Castro, 1976). Given systematically, it causes non-specific stimulation of the reticulo-endothelial system (Halpern *et al.*, 1963) which is regarded as the major mechanism of the anti-tumour action (Jones & Castro, 1977; Milas & Scott, 1978).

Previously our group has shown that i.v. CP causes thrombosis in hepatic, pulmonary and splenic blood vessels in mice, and it was concluded that CP induced disseminated intravascular coagulation (Lampert *et al.*, 1977). This assumption was supported by a subsequent study which showed prolonged thrombocytopenia after i.v. CP (Jones *et al.*, 1977). The present investigations were designed to elucidate the kinetics and mechanisms of the CP-induced coagulopathy in mice.

### MATERIALS AND METHODS

*Animals.*—Age-matched female C57BL/10 ScSn and BALB/c mice, obtained from Olac (Southern) Ltd., were used.

*Corynebacterium parvum.*—A formalin-killed suspension of CP (Wellcome strain CN6134, 7 mg dry weight/ml) was given at a dose of 350 μg. In one experiment this dose was compared to a lower dose of 70 μg. CP was injected i.v., i.p. or s.c.

*Handling of blood and tissues.*—Blood was collected by cardiac puncture during ether anaesthesia. Two samples were taken from each mouse, the first added to 3·8% sodium citrate (1 in 10) and the second to trace amounts of topical thrombin and ξ-amino-caproic acid. Livers and spleens were removed and weighed.

*Blood tests.*—Platelet counts on citrated blood were performed by the method of Brecher & Cronkite (1950). Blood films were made and the haematocrit estimated by standard techniques.

Prothrombin time, activated partial thromboplastin time and fibrinogen level, were determined by established micro-techniques (Dacie & Lewis, 1975).

Fibrin/fibrinogen degradation products (FDP) were determined by the staphylococcal clumping test (Hawiger *et al.*, 1970).

Platelet volume distribution was evaluated electronically by a Coulter Counter ZBI in connection with a Channelyser.

*Platelet turnover and distribution.*—Blood from 45 age- and sex-matched syngeneic donor mice was mixed 1 in 5 with acid–citrate–dextrose (ACD). Platelets were separated by differential centrifugation, labelled with $^{51}$chromium (Dacie & Lewis, 1975) and 0·2 ml of the suspension was injected i.v. into each of 90 mice.

There were 3 groups of recipients: an untreated control group, a group given 350 μg of CP i.v. 2 days earlier, and a group given 350 μg of CP i.v. 7 days earlier. Ten animals from each group were killed and sampled 6, 13 and 22 h after platelet injection.

Blood was collected by cardiac puncture and weighed. Lungs, livers, spleens and kidneys were excised and immediately weighed. The radioactivity of the tissues was determined in a conventional γ-scintillation counter. Activity was expressed as ct/min both in the whole organ and per mg.

*Splenectomy.*—In one study mice were splenectomised 4 weeks before CP injection.

*ξ-aminocaproic acid (EACA).*—EACA (Kabi Vitrum Ltd) was administered immediately before CP as a single i.p. injection of 300 mg/kg body weight, and given thereafter in the drinking water at a concentration of 4 g/l.

*Indomethacin.* — Indomethacin (Merck, Sharp and Dohme) was dissolved in a small volume of ethanol, diluted to the required concentration with normal saline and adjusted to a pH of 7·4. Treatment was started immediately before CP at a dose of 100 μg in 0·2 ml given i.p. and continued twice daily for 2 days. Thereafter a single daily dose of 50 μg was given.

*Statistics.*—Sets of results were compared by the *t* test for 2 samples.

### RESULTS

*Intravenous CP.*—350 μg of CP injected i.v. into C57BL mice caused a coagulopathy (Table I) characterized by severe thrombocytopenia, a significant decrease in plasma fibrinogen, and a significant increase of serum fibrin/fibrinogen degradation products (FDP). There were minor changes in the prothrombin time and activated partial thromboplastin time. Changes were greatest on the 9th day after CP, with recovery by Day 21. There was an accompanying anaemia, and on the 7th day many fragmented red cells were seen on the blood films. On the 7th day the mean platelet corpuscular volume was significantly greater than the controls (8·20 ± 0·68 fl compared to 6·54 ± 0·55 fl, $P < 0{\cdot}05$).

The effects of 350 μg of CP injected i.v. were compared with those of 70 μg. The changes in spleen weight and number of peripheral-blood platelets are shown in Fig. 1. Liver weight followed the same trend as the spleen. Changes were dose dependent, and the development of hepatosplenomegaly correlated with thrombocytopenia.

BALB/c mice given 350 μg of CP i.v. also developed marked hepatosplenomegaly, and experienced a severe coagulopathy identical to that in C57BL mice.

*I.p. and s.c. CP (Table II).*—I.p. in-

TABLE I.—*Effect of 350 μg CP injected i.v. on Day 0 on platelet count, plasma fibrinogen, fibrinogen-degradation products (FDP), prothrombin time (PT), activated partial thromboplastin time (APTT) and packed cell volume (PCV)*

| | Control | Day 7 | Day 9 | Day 12 | Day 21 |
|---|---|---|---|---|---|
| Number of mice: | 12 | 6 | 6 | 6 | 6 |
| Platelet count ($10^9$/l) ± s.d. | 667 ± 47 | 203 ± 56** | 94 ± 27** | 317 ± 42** | 555 ± 46** |
| Fibrinogen (md/dl) ± s.d. | 265 ± 64 | 129 ± 105* | 58 ± 16** | 93 ± 40** | 208 ± 87 |
| FDP (titre) ± s.d. | 4·7 ± 7·1 | 68 ± 26·8 | 145 ± 105* | 20 ± 12·2 | — |
| PT (sec) ± s.d. | 8·6 ± 1·0 | 11·2 ± 1·6* | 10·3 ± 4·5* | 9·0 ± 1·2 | 10·0 ± 1·0 |
| APTT (sec) ± s.d. | 40·1 ± 3·6 | 33·3 ± 3·7* | 37·5 ± 9 | 38·2 ± 3·5 | 45·8 ± 10·1 |
| PCV (%) ± s.d. | 43 ± 1 | 28 ± 5** | 27 ± 3** | 34 ± 2** | 42 ± 2 |

Significance against control $*P < 0{\cdot}01$, $**P < 0{\cdot}001$.

TABLE II.—*Effect of 350 μg CP injected i.p. or s.c. on Day 0 on platelet count and plasma fibrinogen*

| | | Control | Day 7 | Day 12 | Day 21 |
|---|---|---|---|---|---|
| | Number of mice: | 10 | 5 | 5 | 5 |
| I.p. | Platelet count ($10^9$/l) ± s.d. | 1036 ± 194 | 646 ± 311* | 488 ± 91** | 850 ± 408 |
| | Fibrinogen (mg/dl) ± s.d. | 165 ± 50 | 172 ± 45 | 82 ± 42 | 128 ± 24 |
| S.c. | Platelet count ($10^9$/l) ± s.d. | 1036 ± 194 | 824 ± 200 | 1190 ± 167 | 1035 ± 66 |
| | Fibrinogen (mg/dl) ± s.d. | 165 ± 50 | 204 ± 56 | 224 ± 45 | 269 ± 64* |

Significance against control: *$P < 0{\cdot}01$, **$P < 0{\cdot}001$.

FIG. 1.—Changes in spleen weight and platelet count after i.v. injection on Day 0 of either 350 μg (- - - - -), or 70 μg (— — —), of CP. Each point represents the mean for 4 mice with a bar denoting s.e.

FIG. 2.—Platelet turnover after i.v. injection of 350 μg CP. ——, Control ($t_{\frac{1}{2}} = 28{\cdot}5$ h). — — —, 2 days after CP ($t_{\frac{1}{2}} = 29{\cdot}3$ h). - - - - -, 7 days after CP ($t_{\frac{1}{2}} = 14{\cdot}0$ h).

jection of 350 μg of CP into C57BL mice caused similar but less pronounced coagulation changes than i.v.-injected CP. S.c. injection had little effect on the measured variables.

*Platelet kinetics and distribution.*—Platelet kinetics are shown in Fig. 2. The half-life of radiolabelled platelets in control mice was 28·5 h. The half-life was similar 2 days after i.v. CP injection, but by the 7th day had decreased to 14·0 h and indicated rapid disappearance of platelets from the circulation.

Table III shows spleen weight, liver weight and radiolabelled platelet distribution in these mice 22 h after platelet injection. When the distribution of radiolabelled platelets was expressed as ct/min/mg of tissue, CP-treated animals had less

TABLE III.—*The effect of 350 μg of CP on spleen weight, liver weight and radioactivity in spleen and liver 22 h after i.v. injection of $^{51}Cr$ platelets*

| | | Spleen | | | Liver | | |
|---|---|---|---|---|---|---|---|
| Group | No. mice | Organ weight (mg) (mean ± s.d.) | ct/min whole organ (mean ± s.d.) | ct/min/mg (mean ± s.d.) | Organ weight (mg) (mean ± s.d.) | ct/min whole organ (mean ± s.d.) | ct/min/mg (mean ± s.d.) |
| Control | 10 | 100 ± 19 | 7214 ± 605 | 74 ± 13 | 829 ± 155 | 6749 ± 1968 | 8 ± 2 |
| Day 2 after CP | 10 | 180 ± 34** | 10080 ± 2099* | 57 ± 12 | 1077 ± 197 | 10519 ± 1889* | 10 ± 3 |
| Day 7 after CP | 10 | 315 ± 82** | 6843 ± 1967 | 23 ± 8** | 1695 ± 344* | 8762 ± 1877 | 5 ± 2 |

Significance against control: $*P < 0{\cdot}01$, $**P < 0{\cdot}001$.

TABLE IV.—*The effects of splenectomy, ξ-aminocaproic acid and indomethacin treatments on the coagulation changes seen 7 days after i.v. injection of 350 μg CP*

| Treatment | No. mice | Platelet count ($10^9$/l) ± s.d. | Fibrinogen (mg/dl) ± s.d. |
|---|---|---|---|
| Control | 15 | 1061 ± 148 | 182 ± 68 |
| CP | 15 | 172 ± 111** | 104 ± 74* |
| CP + splenectomy | 5 | 151 ± 25** | 140 ± 32 |
| CP + ξ-aminocaproic acid | 5 | 161 ± 33** | 58 ± 21* |
| CP + indomethacin | 5 | 960 ± 85 | 255 ± 117 |

Significance against control: $*P < 0{\cdot}01$, $**P < 0{\cdot}001$.

activity in their spleens than did controls. There was a minor non-significant rise in radioactivity in livers of mice 2 days after CP which decreased by Day 7. Lung and kidney were also examined and contained little radioactivity.

*Treatment.*—Various treatments were given in an attempt to alter the coagulopathy after i.v. CP (Table IV). Splenectomy failed to protect against coagulopathy. EACA-treated animals had enhanced coagulopathy, with lowered fibrinogen and increased thrombosis on histological examination. Indomethacin totally abrogated the coagulation changes. Splenectomy, EACA and indomethacin treatments in normal mice had no effect on the measured variables.

### DISCUSSION

CP has been shown to decrease the number of peripheral-blood platelets (Jones *et al.*, 1977) and we have now demonstrated changes in the plasmatic coagulation system which parallel this thrombocytopenia. There was a marked decrease of plasma fibrinogen, an increase of serum fibrin/fibrinogen degradation products (FDP) and minor changes in the prothrombin and activated partial thromboplastin times. These findings indicate disseminated intravascular coagulation (DIC). The concomitant appearance of many fragmented red cells associated with anaemia suggests microangiopathic haemolytic anaemia, a syndrome considered to be a consequence of intravascular fibrin deposition (Bull & Kuhn, 1970). This explanation of the anaemia following CP would be additional to mechanisms previously described, *i.e.* enhanced phagocytosis and destruction of erythrocytes by the CP-stimulated RES (Cox & Keast, 1974; McBride *et al.*, 1974) and CP-induced autoantibody against red cells (McCracken *et al.*, 1971).

There was no evidence of failure of platelet production, since there was no marrow toxicity (unpublished data) and because the appearance of larger platelets indicated young platelets entering the circulation. This suggested the cause of the thrombocytopenia to be increased peripheral platelet destruction as was confirmed by platelet turnover studies. We considered that the hepatomegaly and splenomegaly might be the sites of this increased platelet destruction. When allowance was made for the 2-fold increase in liver weight and 3-fold increase in spleen weight by the 7th day after i.v. CP, the relative uptake of radioactive platelets by these organs was less than the controls. We found no evidence of active sequestration or passive platelet pooling in the liver, spleen, lung or kidney, and splenectomy failed to prevent any of the coagulation changes. This is surprising since Lampert *et al.* (1977) described thrombi in the liver and spleen of CP-treated mice, but the discrepancy may be explained if the thrombi were both precipitated and removed simultaneously, a phenomenon well known in experimental animals after endotoxin injection (Theiss *et al.*, 1975). Accelerated proteolytic activity is part of the pathophysiology of intravascular coagulation, and its occurrence is supported by the observation that animals treated with the anti-fibrinolytic agent EACA, following injection of CP, had enhancement of the coagulopathy with increased thrombosis.

The correlation between hepato-splenomegaly and coagulation changes suggests that these two phenomena might be mediated by the same mechanism. The organomegaly is associated with an increase in tissue macrophages (McBride *et al.*, 1974; Warr & Šljivić, 1974; Maruyama & Coleman, 1978) and phagocytic activity (Halpern *et al.*, 1963; Adlam & Scott, 1973). Activated macrophages produce various unstable metabolites of arachidonic acid (Brune *et al.*, 1978) including thromboxane $A_2$. This factor can cause intravascular platelet aggregation with subsequent activation of the coagulation system, and its release from macrophages can be inhibited by indomethacin (Brune *et al.*, 1978). Indomethacin treatment of animals following i.v. injection of CP completely prevented the coagulopathy, indicating that enhanced prostaglandin biosynthesis may mediate the coagulation changes.

When the route of administration was altered from i.v., we found that i.p. injection of 350 μg of CP caused similar, but less pronounced, coagulation changes, and s.c. injection had no effect. S.c. CP at this dose has little, if any, anti-tumour action (Sadler & Castro, 1976) suggesting that CP given s.c. does not reach the target macrophages. BALB/c mice injected with CP also developed a severe coagulopathy, demonstrating that this CP effect is not strain specific.

In summary, administration of CP induces a coagulopathy in mice as a consequence of intravascular coagulation with fibrinolysis. It appears that clot-promoting material, possibly derived from the macrophages, initiates this phenomenon. Increased fibrinolytic activity has been found in cancer patients treated with CP (Cederholm-Williams *et al.*, 1978) indicating that our findings might be clinically relevant.

This study was performed in a programme supported by the EORTC Metastasis Project Group and by a grant from the Medical Research Council. H. D. Mitcheson was supported by the Wellcome Foundation.

### REFERENCES

Adlam, C. & Scott, M. T. (1973) Lympho-reticular stimulatory properties of *Corynebacterium parvum* and related bacteria. *J. Med. Microbiol.*, **6**, 261.

Brecher, G. & Cronkite, E. P. (1950) Morphology and enumeration of human blood platelets. *J. Appl. Physiol.*, **3**, 365.

Brune, K., Glatt, M., Kälin, H. & Peskar, B. A. (1978) Pharmacological control of prostaglandin and thromboxane release from macrophages. *Nature*, **274**, 261.

Bull, B. S. & Kuhn, I. N. (1970) The production of schistocytes by fibrin strands (a scanning electron microscope study). *Blood*, **35**, 104.

Cederholm-Williams, S. A., King, A., Allington, M. J., Gill, P. G., Sharp, A. A. & Britton, B. J. (1978). Coagulation and fibrinolysis during the infusion of *Corynebacterium parvum* in man. *Br. J. Cancer*, **37**, 1074.

Cox, K. O. & Keast, D. (1974) Studies of *Corynebacterium parvum*-associated anaemia in mice. *Clin. Exp. Immunol.*, **17**, 199.

Dacie, J. V. & Lewis, S. M. (1975) *Practical haematology*, Vth edn. Edinburgh: Churchill Livingston.

Halpern, B. N., Biozzi, G., Stiffel, C. & Mouton, D. (1966) Inhibition of tumour growth by administration of heat killed *Corynebacterium parvum*. *Nature*, **212**, 853.

Halpern, B. N., Prévot, A. R., Biozzi, G. & 5 others (1963). Stimulation of the phagocytic activity of the reticulendothelial system provoked by *Corynebacterium parvum*. *J. Reticuloendothel. Soc.*, **1**, 77 (French).

HAWIGER, J., NIEWIAROWSKI, S., GUREWICH, V. & THOMAS, D. P. (1970) Measurement of fibrinogen and fibrin degradation products in serum by staphylococcal clumping test. *J. Lab. Clin. Med.*, **75**, 93.

JONES, P. D. E. & CASTRO, J. E. (1977) Immunological mechanisms in metastatic spread and the antimetastatic effects of *C. parvum*. *Br. J. Cancer*, **35**, 519.

JONES, P. D. E., SADLER, T. E. & CASTRO, J. E. (1977) Effect of *Corynebacterium parvum* on peripheral blood platelets. *Br. J. Cancer*, **36**, 777.

LAMPERT, I. A., JONES, P. D. E., SADLER, T. E. & CASTRO, J. E. (1977) Intravascular coagulation resulting from intravenous injection of *C. parvum* in mice. *Br. J. Cancer*, **36**, 15.

McBRIDE, W. H., JONES, J. T. & WEIR, D. M. (1974) Increased phagocytic cell activity and anaemia in *Corynebacterium parvum* treated mice. *Br. J. Exp. Pathol.*, **55**, 38.

McCRACKEN, A., McBRIDE, W. H. & WEIR, D. M. (1971) Adjuvant-induced anti-red blood cell activity in CBA mice. *Clin. Exp. Immunol.*, **8**, 949.

MARUYAMA, Y. & COLEMAN, M. S. (1978) Induction of spleen cell growth and DNA polymerase activity by *Corynebacterium parvum*. *Cancer Res.*, **38**, 1617.

MILAS, L. & SCOTT, M. T. (1978) Antitumour activity of *Corynebacterium parvum*. *Adv. Cancer Res.*, **26**, 257.

PROCTOR, J., RUDENSTAM, C. M. & ALEXANDER, P. (1973) Increased incidence of lung metastases following treatment of rats bearing hepatomas with irradiated tumour cells and the beneficial effect of *Corynebacterium parvum* in this system. *Biomedicine*, **19**, 248.

SADLER, T. E. & CASTRO, J. E. (1976) The effects of *Corynebacterium parvum* and surgery on the Lewis lung carcinoma and its metastases. *Br. J. Surg.*, **63**, 292.

SMITH, S. E. & SCOTT, M. T. (1972) Biological effects of *Corynebacterium parvum*: III, Amplification of resistance and impairment of active immunity to murine tumours. *Br. J. Cancer*, **26**, 361.

THEISS, W., HILGARD, P. & HEYES, H. (1975) Induction of disseminated intravascular coagulation by endotoxin and saline loading in rats: II. Fibrin deposition and removal. *Thrombosis Res.*, **7**, 47.

WARR, G. W. & ŠLJIVIĆ, V. S. (1974) Origin and division of liver-macrophages during stimulation of the mononuclear phagocytic system. *Cell Tissue Kinet.*, **7**, 559.

WOODRUFF, M. F. A. & BOAK, J. L. (1966) Inhibitory effect of injection of *Corynebacterium parvum* on the growth of tumour transplants in isogeneic hosts. *Br. J. Cancer*, **20**, 345.